# Thinner Central Corneal Thickness is Associated with a Decreased Parapapillary Vessel Density in Normal Tension Glaucoma

**DOI:** 10.1155/2022/1937431

**Published:** 2022-03-26

**Authors:** Lan-Hsin Chuang, Yeo-Yang Koh, Henry S. L. Chen, Yun-Hsuan Lin, Chi-Chun Lai

**Affiliations:** ^1^Department of Ophthalmology, Chang Gung Memorial Hospital, Keelung, Taiwan; ^2^College of Medicine, Chang Gung University, Taoyuan, Taiwan; ^3^Department of Ophthalmology, Chang Gung Memorial Hospital, Linkou, Taiwan

## Abstract

This retrospective cross-sectional study, which enrolled 124 normal tension glaucoma (NTG) eyes and 68 healthy eyes as the control, determined the association between central corneal thickness (CCT) and ocular parameters in NTG. CCT was measured using the Pentacam® system, optical coherence tomography angiography (OCT-A) was adopted to measure the peripapillary and macular area VDs, and spatial data were based on the Garway–Heath map as illustrated in OCT-A. Univariate and multivariate linear regressions were used to statistically analyze for associations between CCT and other factors. In this study, the mean age was similar for both the NTG and control groups. The mean CCT of the NTG group was significantly thinner than that of the control group (533.97 ± 33.11 *µ*m vs. 546.78 ± 38.21 *µ*m; *p* = .022). Considering all the factors, CCT negatively correlated with visual field (VF) pattern standard deviation (univariate, *p* = .045). To analyze structural and functional factors separately, we found a significant positive correlation between CCT and whole disc radial peripapillary capillary VD (VDRPC; multivariate, *p* = .019). To analyze the relationship between all factors and sectoral changes in VDRPC, a significant positive correlation was observed between CCT and inferior temporal VDRPC (univariate, *p* = .039) and inferior nasal VDRPC (VDRPC IN; univariate, *p* = .048). In conclusion, this novel study shows that among NTG participants, a thinner cornea correlated with weaker biomechanical properties susceptible to optic nerve tissue displacement, especially in response to mild transient elevation of IOP, leads to compromised ocular microcirculation.

## 1. Introduction

Glaucoma is a spectrum of diseases and is defined as retinal ganglion cell (RGC) apoptosis and progressive glaucomatous optic nerve atrophy. Normal tension glaucoma (NTG) is a subtype of glaucoma in which glaucomatous optic nerve changes and visual field (VF) defects occur but with consistent intraocular pressure (IOP) measurements lower than 21 mmHg. [1] Because there is no IOP elevation, clinical NTG is often overlooked and tends to be diagnosed when it is at the advanced stage. Several Japanese studies have demonstrated a high prevalence of NTG in Asia and showed that biophysical parameters as an indicator for glaucoma progression are essential for early diagnosis. [2–5] Several theories of the pathophysiology of NTG were proposed, consisting of higher sensitivity to normal IOP, vascular dysregulation, an abnormally high translaminar pressure gradient, and a neurodegenerative process due to impaired cerebrospinal fluid dynamics in the optic nerve sheath compartment; the exact etiology of NTG remains controversial to date. [6] The current management of NTG includes IOP reduction by medical, laser, and surgical intervention [7, 8] and IOP-independent management using vascular maintenance and neuroprotection [9].

Thus far, there is no consensus on the role of central corneal thickness (CCT) in hypertensive glaucoma and normotensive glaucoma. According to population-based studies, advanced glaucomatous damage in primary open-angle glaucoma (POAG) is more likely with a thin cornea. [5, 10] Most studies have shown that NTG is associated with thinner CCT when compared with different subtypes of glaucoma. [5, 11–14] However, to evaluate the biomechanical properties of the cornea, one study demonstrated that there was no significant difference in the CCT when compared with NTG, open-angle glaucoma, ocular hypertension, and control. [15] Additionally, corneal hysteresis (CH), measured using an ocular response analyzer, was significantly lower in the NTG group than that in the control group, indicating that the biomechanical properties correlate with glaucoma. [15].

Recently, Zhang et al. [16] demonstrated that patients with glaucoma or ocular hypertension were associated with lower CH and faster retinal nerve fiber layer (RNFL) loss detected by optical coherence tomography (OCT). In this prospective study, CCT (per 100-*µ*m reduction in thickness) was not shown to correlate with RNFL loss during the minimal 2-year follow-up.

One of the pathomechanisms of glaucoma is ocular perfusion deficit with vascular dysregulation. Ocular ischemia, decreased blood flow to the optic nerve, and perfusion pressure have been associated with the pathogenesis and progression of glaucoma. [17–21] Following the development of OCT angiography (OCT-A), the noninvasive and in vivo measurement of the optic disc and macular capillary vessel density (VD) can be easily accessed. [22] The ocular microcirculation of the posterior segment, measured by OCT-A, also illustrates that optic nerve VD is related to glaucoma with high repeatability and reproducibility. [23] Moghimi et al. [24] used OCT-A to demonstrate that the lower baseline optic nerve and macular VD were associated with faster RNFL loss in mild and moderate POAG. The investigators also found that average CCT was a predictor for faster RNFL thickness decline in POAG (0.11 mm/year).

Accordingly, corneal biomechanical properties, including CH and CCT, are strongly related to glaucoma progression. However, at present, there is a lack of research evaluating the correlation between the biomechanical properties and ocular microcirculation in NTG. To explore the predictors of ocular microcirculation and to determine the correlation of parameters of the anterior and posterior segments would be valuable for the early detection of glaucoma because IOP is within the normal range and can easily be underestimated in NTG. Currently, it is unknown whether CCT correlates with the structural and functional parameters simultaneously in NTG. In this study, our goal was to determine whether CCT correlates with peripapillary VD, RNFL thickness, and the VF defect. Secondarily, we aimed to explore the effect of CCT on the spatial characteristics of peripapillary VD.

## 2. Materials and Methods

We conducted a retrospective observational cohort study at Chang Gung Memorial Hospital, Keelung branch, Taiwan, from 2018 to 2020. The study protocol was approved by the Institutional Review Board of Chang Gung Memorial Hospital. Inclusion criteria were as follows: (1) age more than 20 years, (2) open angles on gonioscopy examination, (3) IOP ≤21 mmHg, and (4) glaucomatous disc and peripapillary retina change corresponding to the VF defect. For the control group, we enrolled healthy participants with no history of criteria (2)-(4). In this study, participants with the following conditions were excluded: axial length >26.5 mm; history of corneal, retinal, or lenticular disease; diabetic retinopathy; history of intraocular surgery within 6 months; and Parkinson's disease or Alzheimer's disease.

All participants underwent comprehensive ophthalmologic examination, including best-corrected visual acuity, IOP measurement, slit-lamp biomicroscopy, central corneal thickness and axial length measurement, gonioscopy, dilated fundus examination, optic disc photography, and OCT-A imaging. IOP was measured using non-contact tonometer NT-530P (NIDEK CO. LTD., Japan), CCT was measured using a rotating Scheimpflug camera (Pentacam^®^; Oculus, Optikgeräte, Germany), and axial length was detected using IOLMaster (Carl Zeiss, Jena, Germany).

Optic nerve head and macular area vessel density were obtained and further analyzed through OCT-A (Angio-Vue; Optovue, Inc., Fremont, CA, USA). The OCT-A device used an 840-nm superluminescent diode to perform A-scans for 3 seconds at a rate of 70 000 scans per second. Next, the device performed 2 consecutive B-scans (M-B frames), each containing 304 A-scans, which were captured using the RTVue system. The machine obtained the macular and optic nerve head scan, respectively by a centered volumetric scan on the fovea with an area of 3 mm × 3 mm and on the optic nerve head with an area of 4.5 mm × 4.5 mm.

Using the Angio-Vue software, the vessel density was defined as the percentage of the area occupied with vessels. To calculate the peripapillary disc vessel density, an area defined as a 750-*μ*m wide elliptical annulus extending from the optic disc boundary, calculating the “radial peripapillary capillary (RPC) segment,” and extending from the ILM to the posterior border of the RNFL was used. The AngioDisc report, based on the Garway–Heath map, provided the calculated vessel density of the six sectors of the peripapillary region. For macular vessel density measurement, the OCT-A software segmented the full-thickness retinal scans into “superficial” and “deep” inner retinal vascular plexuses (MVD sup. and MVD deep, respectively). The superficial inner retina scan measured the vasculature in the area between the RNFL and the ganglion cell layer (GCL), while the deep inner retina scan measured the vascular plexuses between the border of the IPL and the inner nuclear layer (INL) and the border of the INL and the outer plexiform layer (OPL).

**Figure 1:**
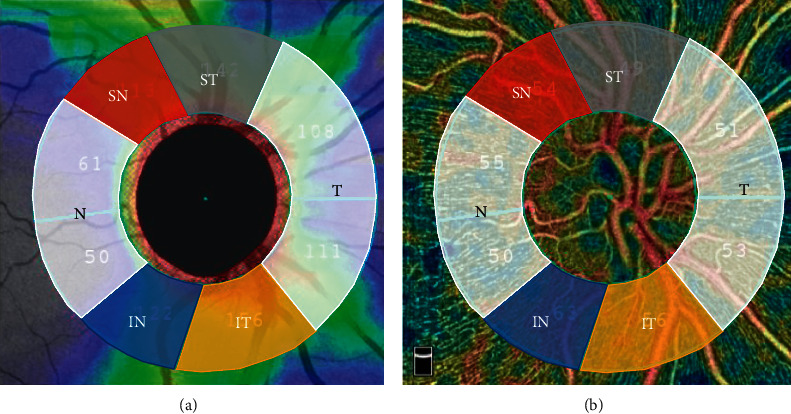
Optical coherence tomography angiography of (a) the retinal nerve fiber layer and (b) radial peripapillary capillary layer of the left eye (OS) with the Garway–Heath map sectors: temporal (T) in white color), superior temporal (ST, in gray color), inferior temporal (IT, in orange color), nasal (N) in white color), superior nasal (SN, in red color), and inferior nasal (IN, in blue color) sectors.

We recorded these data and provided more details for both whole and sectoral peripapillary vessel density and other OCT-A parameters, including retinal nerve fiber layer and ganglion cell complex thickness. Based on the Garway–Heath sector settings, peripapillary disc vessel density and RNFL thickness were initially analyzed as an average value. Six sectors were obtained including the temporal, nasal, superior nasal, superior temporal, inferior nasal, and inferior temporal areas (Figure 1).

Visual function was monitored using the standard automated perimetry (SAP) (Humphrey Field Analyzer II; Carl Zeiss Meditec, Dublin, CA, USA) 30–2 test pattern. Visual filed tests with low reliability, including ≥33% fixation losses, ≥ 10% false positives, ≥ 10% false negatives, and OCT-A with a low signal strength index (<50) were excluded from this study. VF progression was described as the presence of 2 or more points that declined by at least 10 dB from the mean baseline values of these points between the baseline and the final VF examinations. After the VF progression was determined, we further quantified the amount of progression using the decreased mean deviation (MD) slope and pattern standard deviation (PSD) slope (dB/year) during follow-up examinations.

We further classified measurements into structural factors and functional factors. Structural factors included the peripapillary retinal nerve fiber layer thickness (RNFL), ganglion cell complex (GCC) thickness, and ocular microcirculation (whole disc peripapillary vessel density and macular vessel density). However, functional factors consisted of two visual field parameters (mean deviation and pattern standard deviation).

For statistical analyses, descriptive statistics were reported as the mean and standard deviation. Demographic data, such as age, sex, systemic diseases, data of ocular examination, axial length, OCT-A parameters, and VF parameters, were collected during the investigation. Differences in demographics and clinical features between the NTG and control groups were evaluated using the independent sample *t*-test for continuous variables and the chi-square test for categorical variables. To investigate the effect of CCT on ocular structural parameters and ocular functional parameters, univariate and multivariate linear regressions were used for analysis. Statistical analyses were performed using SPSS software, version 20.0 (SPSS, Inc., Chicago, IL, USA). *P* values less than .05 were considered to be statistically significant.

## 3. Results

Overall, 124 NTG eyes from 76 patients and 68 nonglaucomatous eyes from 42 controls were enrolled in our study. The mean age of the study group was 59.11 ± 11.33 years. The demographic data of the NTG cases were similar to those of the controls for age, sex, and incidence of diabetic mellitus and hypertension (Table 1). The central corneal thickness was 10 *µ*m thinner in the NTG group (533.97 ± 33.11 *µ*m) than in the control group (546.78 ± 38.21 *µ*m), which was statistically significant (*p* = .022) (Table 2). The NTG group had a slightly longer axial length compared with the control group (24.56 ± 1.18 mm vs 24.16 ± 1.03 mm; *p* = .016).

In the analysis of ocular microcirculation, the NTG group showed significantly decreased whole disc peripapillary vessel density (VDRPC) compared with the control group (45.51 ± 7.36% vs 50.69 ± 3.52%; *p* < .001). We further divided VDRPC into 6 sectors according to the Garway–Heath sector settings; all sectors were significantly decreased compared with the control group. The superficial and deep macular vessel density in NTG patients were significantly decreased compared with the control group (*p* < .001, *p* = .02, respectively; Table 2). In addition to capillary vascularity measurement, peripapillary RNFL thickness was significantly thinner than that in the control group (84.75 ± 14.29 *µ*m vs 98.15 ± 8.64 *µ*m; *p* < .001). GCC thickness was significantly decreased in the NTG group (Table 2). In addition, evaluation of the visual field test demonstrated that the MD slope of the NTG group was −0.045 ± 1.474 dB/year, and the PSD slope was −0.145 ± 1.031 dB/year.

Because CCT is a parameter of the anterior segment, we have shown that CCT is thinner in the NTG group than in the control group. Next, we evaluated the correlation between CCT and the structural indicators of OCT-A using linear regression to analyze the functional parameters of VF. Univariate regression analysis showed that central corneal thickness negatively correlated with visual field PSD (*p* = .045) in the NTG group (Table 3); age and axial length did not significantly correlate with corneal thickness. In multivariate linear regression of structural factors, there was a significant positive correlation between central corneal thickness and peripapillary vessel density (*p* = .019). By contrast, superficial and deep macular vessel density did not correlate with CCT. In addition, there was no correlation between CCT and RNFL or GCC thickness. The multivariate linear regression of the functional factors showed that CCT negatively correlated with VFPSD (*p* = .007). However, VF progression in terms of VF MD or VF PSD change (dB/year) did not significantly correlate with CCT (Table 3). In the control group, CCT did not significantly correlate with VDRPC or VFPSD when analyzed through linear regression.

We used univariate analysis to analyze 4 sectors of the peripapillary vessel density data according to the default OCT-A Garway–Heath map: superior temporal (ST), superior nasal (SN), inferior temporal (IT), and inferior nasal (IN) sectoral parameters (Figure 1). There was a significant positive correlation between CCT and two peripapillary sectors: inferior temporal sector (VD RPC IT, *p* = .039) and inferior nasal sector (VD RPC IN, *p* = .048) (Table 4). However, CCT was only positively correlated with inferior nasal sector of VDRPC (*P* = .002) when analyzed through multivariate linear regression.

## 4. Discussion

The current study has two principal findings. First, the cornea of NTG patients was thinner compared with their healthy counterparts. Second, a thinner cornea of NTG patients correlated with a decreased peripapillary vessel density, especially at the inferior sector, and poorer visual field results.

Previous population-based studies had shown that NTG is associated with a thinner cornea compared with other subtypes of glaucoma. [5, 11–14] Our retrospective observational study for an Asian population showed that the mean CCT of NTG eyes was 533.97 ± 33.11 *µ*m and 10 *µ*m thinner than the CCT of healthy controls (546.78 ± 38.21 *µ*m). The axial length of the NTG group was slightly longer than that of the control group (24.56 mm vs 24.16 mm). There was no significant difference in the demographic data, including age, sex, and systemic diseases such as diabetic mellitus and hypertension, between the NTG and control groups.

CCT had been previously studied to show the variability in age, sex, refractive status of different populations, and corneal thickness decrease with age. [25–29] A thicker CCT had greater resistance; therefore, it led to an overestimation of IOP when measured through applanation tonometry. CCT and CH were two essential corneal parameters of biomechanical property. Park et al. [30] demonstrated that among NTG participants, there was a significant correlation between the CH and CCT (*r* = 0.44, *p* < .01). In the current study, we measured CCT using a rotating Scheimpflug camera (Pentacam^®^), which can measure the whole corneal surface.

With conventional OCT development, structural parameters, such as peripapillary RNFL and GCC, were useful for diagnosing glaucoma and evaluating its progression. After the development of OCT-A, peripapillary vessel density was another parameter that can be used to evaluate the optic nerve microcirculation quantitatively. Moghimiet et al. [24] found that baseline macular and optic nerve head vessel density were associated with an increased rate of optic nerve degeneration. The investigators also concluded that the average CCT was predictive for faster RNFL thickness decline (0.11 mm/year) in mild and moderate POAG cases. [24] We found that RNFL and GCC thickness was significantly decreased in the NTG group, but CCT did not positively correlate to RNFL thickness or GCC thickness when analyzed using linear regression.

Unlike high-tension glaucoma presented with cornea edema and increased corneal thickness when IOP is elevated, NTG usually had less corneal thickness change and/or IOP fluctuation. At present, there is no consensus regarding the pathomechanism of NTG. The possible theories of NTG pathogenesis included the vascular theory of perfusion deficit and vascular dysregulation. [17, 18] In the new era of OCT-A, measurement of peripapillary and macular vessel density showed good reproducibility and accuracy for the diagnosis of glaucoma. [22, 31] Huan Xu et al. [32] demonstrated that peripapillary vessel density in NTG is significantly lower compared with HTG, whereas the RNFL and full retinal thickness were similar for both NTG and HTG. For the various subgroups of glaucoma, ocular microcirculation data, measured using OCT-A, were more informative than RNFL and GCC thickness. To explore the accessible predictor correlated to the structural and functional changes of NTG is important for early diagnosis and during the longtime care before the irreversible loss of vision.

The mechanism by which thinner CCT predisposed to decreased peripapillary vessel density and glaucomatous optic neuropathy was still unknown. According to the previous literature, several studies had investigated the correlation between corneal biomechanics and the neuroretinal tissue in glaucoma. Burgoyne et al. [33] proposed that even at low IOP, IOP-related stress and strain were biomechanically substantial within the load-bearing connective tissues of the optic nerve head. Mohamed-Noor et al. [34] concluded that for glaucoma subgroups, correlation between CCT (measured using ultrasonic pachymetry) and anterior sclera thickness (measured using ultrasonic biomicroscopy) was found only in NTG, not in OHT, POAG, or healthy controls. The investigators hypothesized that the cornea biomechanically related to ocular tissues, such as sclera and lamina cribrosa; therefore, a thinner cornea reflected a predisposition to glaucoma.

Another study showed that in NTG, CCT positively correlated with sclera thickness and CH. [30] The authors hypothesized that low CH reflected structural weakness against the stress or strain of posterior ocular tissues, leading to an increased risk of VF progression. Owing to the development of OCT, CH and posterior segments can be quantitatively measured and evaluated. Zhang et al. [16] conducted a study of glaucoma patients with 3.8 years of follow-up. Using a multivariate model that accounted for age, race, average IOP, and CCT, it was found that CH affected the rate of RNFL progression, with low CH relating to faster RNFL loss. The investigators hypothesized that CH was related to connective tissue properties and minimizes the IOP change; therefore, lower CH exposed RGC to mechanical strain.

Despite CCT and CH representing the properties of corneal biomechanics, there was no consensus on their role in other parameters related to glaucomatous optic neuropathy among various groups of glaucoma. Jonas and Holbach [35] concluded that in enucleated nonglaucomatous eyes, CCT may not correlate significantly with lamina cribrosa thickness and peripapillary scleral thickness. They suggested that the assumed relationship between CCT and glaucoma susceptibility could not be fully explained by the anatomical similarity between CCT and the optic nerve head. Our study demonstrated that in NTG participants, CCT significantly correlates with the VF PSD (*p* = .045). To avoid the effects of confounding factors, we further analyzed the structural and functional factors separately using multivariate analysis. The result revealed a positive correlation between CCT and peripapillary vessel density among these structural factors (*p* = .019). This may support our hypothesis that under normal IOP (<20 mmHg), a thinner cornea has weaker biomechanical properties; thus, it was unable to dampen or compensate IOP changes, leading to compromised ocular microcirculation. However, we could not find any statistically significant correlation between CCT and RNFL thickness or GCC in NTG. Concerning structural factors, our study demonstrated that the correlation with CCT was stronger than that of RNFL thickness, supporting the vascular theory as the pathomechanism and RPC as being valuable in NTG diagnosis. Functionally, CCT also positively correlated with visual field PSD when analyzed using multivariate analysis, suggesting that a thin cornea strongly related to the deterioration of visual function seen in NTG participants(*P* = .007). In addition, CCT did not correlate with any other OCT-A parameters in the control group.

On the basis of the aforementioned findings, we further analyzed the spatial characteristics of ocular microcirculation stratified by the Garway–Heath sectors map. Using univariate linear regression for all confounding factors and RPC with different sectors, the result showed that CCT positively correlates with inferior nasal (*p* = .039) and inferior temporal side (*p* = .048) peripapillary vessel density in NTG (Table 4). Multivariate regression for structural parameters demonstrated a significant correlation between inferior nasal RPC and thinner cornea (*p* = .002). This is the first study to show that the CCT, a parameter of the anterior segment, positively correlated with spatial peripapillary capillary vessel density in NTG.

Currently, there is no hypothesis to explain the correlation between CCT and RPC with spatial characteristics. One study aimed to investigate the anterior segment parameter measurements as a surrogate for optic nerve displacement upon IOP increase. It was found that the degree, direction, and spatial change of cup movement are associated with IOP and corneal thickness but not corneal hysteresis.^34^ The investigators found that a thinner cornea results in the optic disc moving more superiorly during mildly elevated IOP (*R*^2^ = 0.35, *p* = .007). [36] As we have demonstrated that the sectoral characteristics of CCT negatively correlate with peripapillary capillary dropout, we hypothesize that a thinner cornea may directly provoke any change in IOP and increase the susceptibility of glaucoma. Consistently, our results agree with the findings of Bedggood et al. [36] which show that a thinner CCT correlates with a decrease in inferior sectoral peripapillary vessel density in NTG.

Using OCT-A, Xu et al. [32] demonstrated a significantly lower VD in NTG compared with HTG. By contrast, Scripsema et al. [37] showed that in early and moderate glaucoma, patients with HTG had lower vessel density compared with patients with NTG, although the cause of the difference in vessel density was not fully understood. Similarly, our results also demonstrated that a thinner cornea is structurally consistent with the correlation of decreased vessel density with spatial characteristics; however, the lack of correlation with RNFL implied the importance of VDRPC decrease in NTG. Our study used a Garway–Heath map to segment the AngioDisc report and showed that the inferior nasal sector predominantly decreased in NTG and positively correlated to CCT when analyzed using univariate linear regression (Table 4). Multiple linear regression analysis of CCT structural factors also resulted in positive correlations with inferior nasal sector peripapillary vessel density. In addition, for early glaucoma, it has been reported that peripapillary vessel density is lower in the inferior temporal and superior temporal sectors with corresponding focal RNFL defects. [38] The discrepancy between studies in vessel density spatial change for AngioDisc and RNFL of optic nerve head map in NTG should be noted; the differences can be attributed to the differences in the OCT-A software for sector definitions. A thinner cornea with sectoral changes in VDRPC could help in the diagnosis of NTG.

There are several limitations to our study, such as the small sample size and the retrospective nature of the study that used a single OCT-A machine for measuring peripapillary VD, RNFL, and GCC. A cross-sectional observation study could not determine the reason for a thinner cornea leading to peripapillary capillary dropout. A longitudinal change associated with visual function status is required to clarify the importance of CCT in NTG progression.

## 5. Conclusions

In conclusion, central corneal thickness measurement is an accessible examination in various levels of the healthcare system. In clinical practice, NTG patients presented with thinner cornea may have decreased ocular microcirculation and poorer visual field results than their counterparts with thicker cornea. Close follow-up and meticulous examinations are necessary for these patients.

## Figures and Tables

**Table 1:** Demographics of the NTG and control groups.

Variable	NTG (*n* = 76)	Control (*n* = 42)	*p* value^a^
Age, years (mean ± SD)	59.11 ± 11.33	56.83 ± 12.66	0.319
Sex ratio (Male:Female)	1.3 (43 : 33)	2.0 (28 : 14)	0.284
Diabetes mellitus, *n* (%)	16 (21.1%)	8 (19.0%)	0.796
Hypertension, *n* (%)	21 (27.6%)	16 (38.1%)	0.241

^a^Comparison of two groups using independent t-test or chi-square test.

**Table 2:** Clinical parameters of the NTG and control groups.

Variable	NTG (*n* = 124)	Control (*n* = 68)	*p* value^a^
*CCT (µm)*	533.97 ± 33.11	546.78 ± 38.21	0.022
*AL (mm)*	24.56 ± 1.18	24.16 ± 1.03	0.016
*VF MD (dB)*	−6.45 ± 6.70	−1.45 ± 2.75	<0.001
*VF PSD (dB)*	6.65 ± 4.44	2.97 ± 2.59	<0.001
*VD RPC (%)*	45.51 ± 7.36	50.69 ± 3.52	<0.001
*VD RPC SN (%)*	43.16 ± 7.30	46.47 ± 5.53	0.001
*VD RPC ST (%)*	48.32 ± 8.56	52.83 ± 4.46	<0.001
*VD RPC IN (%)*	43.33 ± 9.89	49.95 ± 5.07	<0.001
*VD RPC IT (%)*	44.73 ± 14.17	55.79 ± 5.89	<0.001
*MVD sup (%)*	41.10 ± 5.70	44.76 ± 4.01	<0.001
*MVD deep (%)*	48.22 ± 4.69	50.30 ± 3.84	0.002
*RNFL (µm)*	84.75 ± 14.29	98.15 ± 8.64	<0.001
*RNFL SN (µm)*	94.39 ± 21.81	106.56 ± 16.88	<0.001
*RNFL ST (µm)*	112.73 ± 27.04	134.21 ± 14.05	<0.001
*RNFL IN (µm)*	91.15 ± 20.22	108.19 ± 19.59	<0.001
*RNFL IT (µm)*	107.24 ± 30.60	137.54 ± 17.94	<0.001
*GCC (µm)*	85.10 ± 12.12	94.25 ± 5.81	<0.001

^a^Comparison of two groups using independent t-test. CCT: central corneal thickness; AL: axial length; VF MD: visual field mean deviation; VF PSD: visual field pattern standard deviation; VD: vessel density; RPC: radial peripapillary capillary network; SN: superior nasal sector; ST: superior temporal sector; IN: inferior nasal sector; IT: inferior temporal sector; MVD sup: superficial macular vessel density; MVD deep: deep macular vessel density; RNFL: retinal nerve fiber layer; GCC: ganglion cell complex.

**Table 3:** Confounding factors associated with central corneal thickness in the NTG group evaluated via linear regression analysis.

Variable	Coefficient (95% CI)	*p*value^a^
Age	−0.129 (−0.921, 0.145)	0.152
Sex	−0.003 (−12.048, 11.669)	0.975
DM	0.051 (−10.061, 18.171)	0.571
HTN	−0.073 (−18.638, 7.786)	0.418
AL	0.003 (−4.949, 5.139)	0.970
VF MD	−0.002 (−0.896, 0.876)	0.982
VF MD change	0.117 (−0.716, 2.669)	0.255
VF PSD	−0.181 (−2.662, -0.032)	**0.045**
VF PSD change	0.033 (−1.905, 2.628)	0.752
VD RPC	0.134 (−0.194, 1.404)	0.137
MVD Sup whole	−0.030 (−1.216, 0.864)	0.738
MVD Sup parafovea	−0.031 (−1.158, 0.816)	0.732
MVD Deep whole	−0.015 (−1.372, 1.159)	0.868
MVD Deep parafovea	0.028 (−0.975, 1.338)	0.757
RNFL	0.124 (−0.125, 0.700)	0.170
GCC	0.072 (−0.292, 0.685)	0.428

^a^Analyzed with univariate linear analysis. DM: diabetes mellitus, HTN: hypertension; AL: axial length; VF MD: visual field mean deviation; VF PSD: visual field pattern standard deviation; VD: vessel density; RPC: radial peripapillary capillary network; MVD sup: superficial macular vessel density; MVD deep: deep macular vessel density; RNFL: retinal nerve fiber layer; GCC: ganglion cell complex.

**Table 4:** Sectoral VDRPC associated with CCT in the NTG group evaluated via linear regression analysis.

Variables	Coefficient (95% CI)	*p* value^a^
VD RPC ST	0.091 (−0.327, 1.005)	0.316
VD RPC SN	0.130 (−0.219, 1.403)	0.151
VD RPC IT	0.186 (0.022, 0.846)	**0.039**
VD RPC IN	0.178 (0.004, 1.185)	**0.048**

^a^Analyzed with univariate linear analysis. VD: vessel density; RPC: radial peripapillary capillary network; ST: superior temporal sector; SN: superior nasal sector; IT: inferior temporal sector; IN: inferior nasal sector.

## Data Availability

The data used to support the findings of this study are restricted by the Institutional Review Board of Chang Gung Memorial Hospital in order to protect patient privacy. Data are available from Dr. Lan-Hsin Chuang (e-mail address: lanhsin.chuang@gmail.com) for researchers who meet the criteria for access to confidential data.
